# Initial psychometric properties of the national entitlement scale and its association with social dominance orientation and psychological sense of global community

**DOI:** 10.1186/s40359-026-04826-6

**Published:** 2026-06-04

**Authors:** Zafer Güney Çağış

**Affiliations:** Department of Psychology, Inonu University, 44000 Battalgazi/Malatya, Türkiye

**Keywords:** National Entitlement Scale, Entitlement, Social dominance, Psychological sense of global community

## Abstract

**Background:**

Many people tend to think that the social groups they belong to deserve more than other social groups, and for some, this tendency is more pronounced. National entitlement represents this tendency in national identities and refers to one’s unrealistic belief that one’s nation is privileged and entitled to more than other nations. In other words, national entitlement reflects one’s belief that one’s nation should achieve various gains, regardless of whether the nation actually deserves them. To date, no comprehensive, valid, and reliable scale exists to measure national entitlement. Therefore, the primary purpose of this research is to adapt the Psychological Entitlement Scale to the national level and to examine its relationship with social dominance orientation and psychological sense of global community.

**Methods:**

The participant group included 335 young and middle-aged Turkish adults (55.2% men; *M* = 29, *SD* = 7.27). The factor structure of the 8-item National Entitlement Scale was examined using exploratory factor analysis (EFA) and confirmatory factor analysis (CFA). The relationship between national entitlement, social dominance orientation (SDO), and psychological sense of global community (PSGC) was examined using Pearson correlation analyses and the PROCESS macro (Model 4).

**Results:**

Factor analyses suggested a one-factor structure with high reliability for the National Entitlement Scale. Pearson correlation analysis revealed that national entitlement was positively correlated with SDO and negatively correlated with PSGC. SDO was also negatively correlated with PSGC. Importantly, PSGC significantly mediated the relationship between SDO and national entitlement.

**Conclusions:**

The results indicate that the 8-item National Entitlement Scale is psychometrically valid and reliable. Moreover, the findings highlight the role of PSGC in the relationship between SDO and national entitlement.

## Background

Psychological entitlement is a component of narcissism and is the expression of the belief that one should achieve gains regardless of whether one deserves them [1]. Individuals high in psychological entitlement expect special treatment in an egocentric way, believe that they are exempt from everyday social demands, and want events to begin, continue, and end as they wish. These individuals aim to obtain resources that they do not deserve, even at the expense of others, and act in pursuit of this goal [2]. They hold self-image-focused goals [3] and tend to act hostilely and aggressively to protect and increase their self-image [1, 4]. Karim [5] found that psychological entitlement is positively associated with Machiavellianism, narcissism, and psychopathy. Psychological entitlement, which is positively correlated with such adverse outcomes at the individual level, may also emerge at the national level and requires in-depth analysis, especially in social and cultural contexts. Accordingly, in this study, the concept of psychological entitlement was examined at the national level. For this purpose, in the present study, the Psychological Entitlement Scale developed by Campbell et al. [1] was adapted to the national level, and the mediating role of the psychological sense of global community (PSGC) in the relationship between social dominance orientation (SDO) and national entitlement was tested, controlling for age, gender, and socioeconomic level.

The Social Dominance Theory [6] provides a critical theoretical framework for understanding the origins of social structures and inequalities between groups. The theory focuses on the causes of social hierarchies, emphasizing that people tend to form group-based systems of dominance. According to the theory, age and gender groups and hierarchies between them are present across all societies. Similarly, arbitrary systems of social hierarchy emerge in social contexts where sustainable economic surpluses exist, and these hierarchies not only determine social status but also shape ideologies and behaviours that reinforce the perpetuation of inequality [6, 7]. For example, the theory reveals how inequality between genders operates as part of broader systems of dominance. According to Social Dominance Theory, men generally have more economic and social power, and men tend to keep women in a lower status to maintain their advantageous position [6]. In addition, men exhibit higher levels of social dominance orientation than women, which further reinforces the continuity of the hierarchy between genders [8].

Although group-based oppression has many consequences, the Social Dominance Theory suggests that SDO largely affects the attitude toward the outgroup. SDO is defined as the desire of individuals to maintain inequalities between groups and their tendency to endorse and support such inequalities [9, 10]. SDO predicts ideologies that cause negative intergroup attitudes, such as sexism and racism [11]. Individuals high in SDO perceive group-based disparities as legitimate and natural, and prefer hierarchical social structures over egalitarian social structures [6]. For this reason, they tend to support hierarchy-enhancing legitimizing myths (e.g., sexism, nationalism) rather than hierarchy-attenuating myths (e.g., social democracy, egalitarianism, human rights). In addition, these people believe that the social groups they belong to are inherently superior to others [9, 12]. Pratto et al. [8] revealed that SDO was significantly and positively correlated with patriotism, nationalism, chauvinism, and cultural elitism, reflecting an attitudinal bias in favour of the national ingroup. Similarly, subsequent studies have also indicated that SDO is positively correlated with ethnocentrism [13], racism [14, 15], nationalism [16], patriotism [17–19], and national entitlement [20].

National identity encompasses the feelings of belonging and loyalty towards any nation-state, and, like all other social identities, it also influences intergroup relations [21]. While national identity is positively associated with favourable attitudes toward subnational identities within the nation (e.g., ethnic or regional subgroups) [22, 23], it can also be associated with negative attitudes toward members of other nations [24]. The intensity of this negative attitude is associated with the strength of national identification [25].

Most people tend to positively evaluate the social groups they belong to [26], but this perception can sometimes become exaggerated. In other words, narcissistic tendencies at the individual level can also emerge at the group level. Studies have revealed that people may hold unrealistically positive beliefs about their national identity, and such a perception is associated with negativity, negative feelings, prejudice, and aggression toward the outgroup [27–30].

Each nation tends to see itself as somehow superior or entitled over others, although the intensity of this tendency varies across contexts and individuals. National entitlement refers to an individual’s unrealistically positive perception of their nation, characterized by an unrealistic belief that their nation deserves to obtain special advantages or significant gains. National entitlement is similar to collective narcissism in that both concepts emphasize social identity and its psychological consequences. However, the two differ in their origins, core emphases, and modes of operation. Both national entitlement and collective narcissism focus on a strong sense of identification with the group (the nation in national entitlement and any salient group in collective narcissism) and the belief that the group is exceptional and superior. Moreover, individuals high in both national entitlement and collective narcissism tend to be emotionally reactive to perceived threats or criticism of their group [20, 29].

However, national entitlement reflects the belief that the nation is inherently entitled to certain privileges due to its historical, cultural, or political significance. In contrast, collective narcissism is based on an inflated yet insecure belief in the group’s grandiosity and requires continual external approval and admiration [29]. Moreover, while psychological entitlement focuses specifically on claims of entitlement at the nation-state level, collective narcissism can emerge at any group level, such as religious, ethnic, or ideological groups [31].

National identity is also conceptually linked to constructs such as ethnocentrism and nationalism, since all of them include evaluations of the in-group’s relationship with out-groups. Ethnocentrism is the tendency to view one’s own in-group members as virtuous and superior, whereas out-group members are considered inferior, and to prioritize in-group interests in various areas [32]. On the other hand, nationalism refers to an ideology that prioritizes a person’s loyalty to their nation over other social identities such as gender or religion, emphasizes national identity, and advocates for the protection of the nation [33]. However, national entitlement differs from these constructs in terms of its particular emphasis on perceived entitlement. National entitlement does not only reflect a preference for one’s own group. It can be argued that it generally reflects a belief that one’s nation is inherently entitled to special treatment, privileges, or advantages, independent of objective conditions (Campbell, et al., [1]; Cai & Gries [20]. Therefore, national entitlement can be considered a more specific and entitlement-focused belief that may be related to ethnocentric or nationalist orientations but cannot be equated with them. Accordingly, although national entitlement may theoretically be associated with collective narcissism, ethnocentrism, and nationalism, in this study, national entitlement has been conceptualized as a distinct construct from these structures.

Given its conceptual distinctiveness as well as its overlap with related concepts including collective narcissism, ethnocentrism, and nationalism, national entitlement may be associated with negative intergroup factors. In fact, although studies on national entitlement are limited, Cai and Gries [20] have found that national entitlement has a positive correlation with negative attitudes toward out-groups.

The psychological sense of community refers to a perceived interdependence with others and the desire to sustain this interdependence [34]. In other words, it involves recognizing shared similarities and a sense of connection. Therefore, this sense is positively related to egalitarianism by reducing the potential adverse effects of egocentrism [35]. It fosters prosocial behavior and cooperation, particularly among group members [36], by encouraging individuals to prioritize collective interests over personal ones [37].

Although the psychological sense of community is often seen as relatedness between people living in a limited geographical area, such as a neighbourhood and a town, PSGC extends beyond these geographical boundaries [36, 38]. Thus, it refers to a sense of connection with people around the world with whom one has no direct or indirect contact [39]. This sense encompasses people’s social identities, including sexual, religious, or national identities. For this reason, PSGC can promote concern for the welfare of all people and is associated with prosocial behaviors, a sense of global social responsibility, global activism, and empathy [39]. In addition, individuals high in PSGC tend to hold positive attitudes toward asylum seekers [40]. Hackett et al. [41] also indicated that PSGC has a significant role in the relationship between people’s values and human rights concerns and behavior.

### Research gap and rationale

Despite growing scholarly interest in entitlement-based beliefs (e.g [42, 43]), , research on entitlement at the national level remains limited. Cai and Gries [20] provided the first empirical conceptualisation of national entitlement concept by adapting 6 items from the Psychological Entitlement Scale; however, they only examined the reliability of the measure and did not evaluate its broader psychometric properties. To date, a comprehensive validity study of the expanded national entitlement scale has not been conducted, yet theoretically, it may be associated with significant intergroup outcomes. This gap limits the ability of researchers to examine national level entitlement in various sociocultural contexts.

A second gap concerns the mechanisms through which SDO may contribute to national level entitlement beliefs. While previous studies have indicated that SDO predicts hierarchy-enhancing ideologies and beliefs in ingroup superiority [8–20], it remains unclear how these dominance-oriented tendencies translate into exaggerated beliefs about national entitlement. Moreover, no studies have examined whether PSGC, an egalitarian, globally oriented sense of connection, can buffer SDO’s effects on national entitlement. Addressing these gaps is essential for understanding both the structure of national entitlement concept and the psychological processes underlying its construction.

### The current study

To address these gaps, the present study had two main aims. First, it sought to examine the psychometric properties of an 8-item National Entitlement Scale, readapted from the Psychological Entitlement Scale developed by Campbell and colleagues [1], providing a more comprehensive measurement than previous adaptations [20]. Second, the current study aimed to examine whether PSGC mediates the relationship between SDO and national entitlement, based on evidence indicating that SDO supports hierarchical intergroup structures [8–12], whereas PSGC enhances egalitarianism and concern for people all over the world [35–44]. By synthesizing existing literature, this research provides insights into how hierarchy-reinforcing orientations shape beliefs about national superiority and the psychological conditions under which these beliefs may be attenuated. Accordingly, the hypotheses of the current study were as follows:

- H1: The National Entitlement Scale shows good internal consistency reliability.
- H2: The National Entitlement Scale yields a one-factor solution.
- H3: SDO is positively correlated with the National Entitlement Scale, whereas it is negatively correlated with PSGC.
- H4: PSGC is negatively correlated with the National Entitlement Scale.
- H5: PSGC has a mediating role in the relationship between SDO and national entitlement after controlling for age, gender, and socioeconomic level.

## Research methodology

### Participants

The participant group of the present study consisted of 335 young and middle-aged Turkish adults. The participants were aged between 18 and 50 (*M* = 29, *SD* = 7.27). Of the participants, 150 (44.8%) were women and 185 (55.2%) were men. Most of them (74.3%) had a Bachelor’s degree. Regarding socioeconomic status, 112 participants (33.4%) were classified as low socioeconomic status, 172 (51.4%) as middle socioeconomic status, and 51 (15.2%) as high socioeconomic status (see Table 1).

**Table 1:** Characteristics of the participant group (*N* = 335)

	Group	*n*	%
Gender	Women	150	44.8
	Men	185	55.2
Education level	High school	42	12.5
	Higher school	249	74.3
	Master and upper	44	13.2
Socioeconomic level	Low	112	33.4
	Middle	172	51.4
	High	51	15.2

The participant group was recruited using convenience sampling, and it was not intended to constitute a nationally representative sample. Furthermore, such sampling strategies are commonly used in psychological research for initial validity studies of psychological constructs and attitudinal measures (e.g [45]), .

To provide greater clarity about the characteristics of the participant group, the demographic characteristics were compared with national population statistics in Türkiye. When comparing the demographic characteristics of the participant group with those of the general population in Türkiye, the gender distribution largely corresponds to national statistics. According to the Turkish Statistical Institute [46], approximately 50.02% of the population is men and 49.98% is women, indicating a balanced representation in the participant group. Similarly, the age range of the participant group (18–50 years) broadly encompasses young and middle adulthood, which is particularly appropriate for evaluating the attitudinal and ideological structures examined in this study.

With regard to socioeconomic status, the distribution of the participant group in the current study (33.4% low, 51.4% middle, and 15.2% high socioeconomic status) is closely consistent with the national statistics in Türkiye (35.3% low, 52.6% middle, and 12.1% high socioeconomic status; [47]). This pattern suggests a largely similar socioeconomic distribution, with a slight overrepresentation of individuals from higher socioeconomic levels.

### Measures

#### Psychological sense of global community scale

Malsch [39] adapted the Sense of Community Index [37] to assess PSGC. The PSGC Scale has a unidimensional structure and includes 12 items (e.g., “Despite other differences, people all over the world share similar values”) and is rated on a 5-point Likert-type scale (1 = Strongly disagree, 5 = Strongly agree). In some subsequent studies [41], it was observed that only these 4 items of the scale were used: “People all over the world have a shared fate”, “I feel a sense of connection to people all over the world, even if I don’t know them personally”, “At the end of the day, all people living in the world want the same things”, and “I feel a sense of belonging to a human or world community, one that extends beyond where I live and includes more than just people I know”. In this study, all 12 statements were used by the researcher as suggested by Malsch [39], and it was observed that the scale had a single-factor structure with an acceptable reliability value (*α* = 0.72).

#### Social dominance orientation scale

The SDO Scale by Sidanius et al. [9] consists of 16 items, rated on a 7-point Likert-type scale (1 = Strongly disagree, 7 = Strongly agree). An example item is “Inferior groups should stay in their place”. The lowest score that can be obtained from the general scale is 16, and the highest score is 112. High scores indicate greater SDO. Many previous studies [8] have suggested that the scale has a single factor and a high internal consistency coefficient above 0.80. The Cronbach’s alpha was 0.79 in the present study.

#### National entitlement scale

The National Entitlement Scale was adapted by the researcher from the Psychological Entitlement Scale developed by Campbell and colleagues [1]. The scale includes 8 items, including “I honestly feel that Türkiye is more deserving than other countries” and “Great things should come to Türkiye”. Responses are given on a 7-point Likert-type scale ranging from 1 (Strongly disagree) to 7 (Strongly agree) (see the Appendix for the Turkish and American versions of the National Entitlement Scale). High scores indicate a high level of national entitlement. In the present study, the scale indicated strong internal consistency, with a Cronbach’s alpha value of 0.89.

### Procedures

In line with the primary purpose of the study, the items of the Psychological Entitlement Scale developed by Campbell et al. [1] were modified for the national context by two scholars who are experienced in the relevant literature. Except for the item “If I were on the Titanic, I would deserve to be on the first lifeboat!”, 8 items of the Psychological Entitlement Scale were revised for the national context. Thus, “I honestly feel I’m just more deserving than others” was adapted to “I honestly feel that Türkiye is just more deserving than other countries”; “Great things should come to me” was adapted to “Great things should come to Türkiye”; “I demand the best because I’m worth it” was adapted to “I demand the best for Türkiye because Türkiye is worth it”; “I do not necessarily deserve special treatment” was adapted to “Türkiye does not necessarily deserve special treatment in the international community”; “I deserve more things in my life” was adapted to “Türkiye deserves more than what it has”; “People like me deserve an extra break now and then” was adapted to “Countries like Türkiye deserve an extra break now and then”; “Things should go my way” was adapted to “Things should go Türkiye’s way”; “I feel entitled to more of everything” was adapted to “Türkiye is entitled to more of everything”. The final items are presented in Table 2 and Appendix 1.

**Table 2:** Descriptive statistics, reliability, and factor loadings of the National Entitlement Scale

Item	Participant group I (*n* = 168)					Participant group II (*n* = 167)				
	M	SD	IC	CD	EFA loadings	M	SD	IC	CD	CFA loadings
1. I honestly feel that Türkiye is just more deserving than other countries.	4.60	2.02	0.68	0.88	0.77	4.48	2.07	0.71	0.86	0.80
2. Great things should come to Türkiye.	4.11	1.99	0.76	0.87	0.83	4.01	2.02	0.76	0.87	0.85
3. I demand the best for Türkiye because Türkiye worth it.	5.10	1.88	0.66	0.88	0.75	5.10	1.85	0.67	0.87	0.74
4. Türkiye does not necessarily deserve special treatment in the international community. (R)	3.63	1.90	0.46	0.90	0.55	3.84	2.22	0.39	0.90	0.39
5. Türkiye deserves more than what it has.	5.15	1.90	0.71	0.88	0.80	5.29	1.78	0.63	0.87	0.72
6. Countries like Türkiye deserve an extra break now and then.	4.38	1.83	0.65	0.88	0.74	4.40	1.94	0.70	0.86	0.69
7. Things should go Türkiye’s way.	3.91	1.98	0.70	0.88	0.78	3.96	1.97	0.65	0.87	0.67
8. Türkiye is entitled to more of everything.	4.38	2.04	0.74	0.87	0.81	4.33	2.01	0.73	0.86	0.75
	*R²* = 57.55%					*R²* = 56.30%				
	*α* = 0.89					*α* = 0.88				

*α* Cronbach’s alpha, *M* mean, *SD* Standard deviation, *IC* Corrected item-total correlation, *CD* Cronbach’s alpha if item deleted, *R* Reverse-coded item

Then, the data of the current study were collected online from volunteers using Google Forms in November 2023, after ethical approval was obtained from Toros University Scientific Research and Publication Ethics Board ([2022]125). In this study, a convenience sampling strategy was used, and the survey link was distributed through social media platforms (e.g., Instagram) and messaging applications (e.g., WhatsApp). To be eligible for the study, participants had to be over 18 years old, literate, have access to the internet, and be able to use the application.

All procedures conducted in this study were conducted in compliance with the 1964 Helsinki Declaration, revised in 2008, and similar ethical standards. Participants were briefed about the purpose of the study and informed that their participation was voluntary. They were also assured that their responses would remain confidential and anonymous and that they could withdraw at any stage without any negative consequences. A link containing the informed consent form of the survey on the first page and scales on the other pages was sent to the participants, and all participants provided informed consent before taking part in this study. Completing the survey took approximately 15–20 min.

### Data analysis

Firstly, the normality assumption of the variables was examined by skewness and kurtosis values. Then, consistent with previous research [48], the data were randomly divided into two subgroups to reveal the structure of the National Entitlement Scale. Exploratory factor analysis (EFA) was executed on the first subgroup (*n* = 168) to identify the underlying factor structure of the scale. Subsequently, confirmatory factor analysis (CFA) was executed on the second subgroup (*n* = 167) to determine whether the factor structure identified in the EFA would replicate. Independent samples t-tests revealed no significant differences between the two subgroups on the study variables. Therefore, all subsequent analyses were run on the overall participant group (*N* = 335). Afterward, Pearson correlation analyses were conducted to explore the relationships between the variables. To examine the hypothesized mediation model, the PROCESS macro (Model 4) [49] was executed. The mediation analysis results were reported using squared multiple correlations (*R*^2^) and unstandardized regression coefficients (*β*). Additionally, indirect effects were determined using the bootstrap approach with 5,000 resamples to obtain 95% confidence intervals [50]. SPSS v20 for Windows was used for all analyses.

## Results

### Preliminary analyses

Before conducting the analyses to test the main hypotheses, the dataset was screened for both univariate and multivariate outliers. Among the data collected from 341 participants, 6 participants had z-scores outside the range of -2.5 to + 2.5 and were therefore excluded, as recommended by Field [51]. Subsequently, multivariate outliers were examined using the Mahalanobis distance based on the chi-square distribution (*p* < .001); however, no cases exceeded the critical cut-off value, indicating the absence of multivariate outliers [52]. Thus, the subsequent analyses were carried out with 335 participants. Then, the preliminary analysis findings demonstrated that the skewness values ranged from −0.36 to 0.24, and the kurtosis values ranged from −0.68 to −0.14, suggesting that all variables had a reasonably normal distribution [53] (see Table 3).

**Table 3:** Descriptive statistics, Cronbach’s alphas, and correlations of the study variables (*N* = 335)

	Mean	SD	Skew	Kurt	α	1	2	3
National entitlement	4.41	1.47	−0.36	−0.68	0.89	-	0.24**	−0.22**
Social dominance	2.63	0.82	0.23	−0.48	0.79		-	−0.44**
Psychological sense of global community	3.60	0.51	0.24	−0.14	0.71			-

*α* Cronbach alpha, *M* mean, *SD* Standard deviation, *Skew* Skewness, *Kurt* Kurtosis

** p < .01

Table 2 presents the descriptive statistics, item-total correlation coefficients, and internal consistency coefficients for the National Entitlement Scale in both subgroups. Item-total correlation ranged from 0.46 to 0.76 in the first subgroup and from 0.39 to 0.76 in the second subgroup. The reliability of the National Entitlement Scale was interpreted using Cronbach’s alpha, which was 0.89 for the first subgroup, 0.88 for the second subgroup, and 0.89 for the total participant group.

As a result of the EFA conducted on the first participant group (*n* = 168), the dataset was found to be appropriate for factor analysis (KMO = 0.90; Barlett Sphericity, χ2 [*df* = 28] = 656.69; *p* < .001). The EFA revealed that one eigenvalue was above 1.00, and this factor accounted for 57.56% of the total variance. As shown in Table 2, factor loadings ranged from 0.55 (item 4) to 0.83 (item 2). To provide a more empirically justified criterion for factor retention, a parallel analysis was also run. The findings of the parallel analysis confirmed the retention of a single factor, as the first empirical eigenvalue exceeded the random eigenvalue (95% criterion) [54, 55].

Subsequently, CFA was run on the second subgroup (*n* = 167) to examine whether the one-factor structure obtained in the EFA would be confirmed. The initial goodness-of-fit indices indicated that the one-factor structure yielded a weak fit to the dataset (χ2 [*df* = 20] = 74.06, *p* < .001, χ²/df = 3.70, NFI = 0.89, CFI = 0.92, GFI = 0.90, AGFI = 0.81 RMSEA = 0.128, SRMR = 0.05). Based on the modification indices, the covariances were added between item 2 (“Great things should come to Türkiye”) and item 3 (“I demand the best for Türkiye because Türkiye is worth it”), between item 2 (“Great things should come to Türkiye”) and item 8 (“Türkiye is entitled to more of everything”), and between item 4 (“Türkiye does not necessarily deserve special treatment in the international community”) and item 7 (“Things should go Türkiye’s way”), as these item pairs demonstrated overlapping conceptual content. Following this procedure, the model indicated a good fit (χ2 [*df* = 17] = 34.71, *p* < .01, χ²/df = 2.04, NFI = 0.95, CFI = 0.97, GFI = 0.95, AGFI = 0.90, RMSEA = 0.079, SRMR = 0.04), in line with recommended cutoff criteria [56–58]. Taken together, findings of EFA, parallel analysis, and CFA indicated that a one-factor model was suitable for the National Entitlement Scale.

Independent samples t-tests were performed to investigate whether national entitlement, SDO, and PSGC differed by gender. The results indicated that SDO and PSGC significantly differed by gender. Specifically, men (*M* = 2.72, *SD* = 0.84) reported higher SDO scores than women (*M* = 2.53, *SD* = 0.80) (*t* (333) = -2.10, *p* = .036). In contrast, women (*M* = 3.68, *SD* = 0.47) reported higher PSGC scores than men (*M* = 3.55, *SD* = 0.55) (*t* (332) = 2.17, *p* = .031).

Subsequently, one-way ANOVA was run to examine whether the variables differed by educational status. The results revealed significant differences in national entitlement (*F* = 3.86, *p* = .004) and SDO (*F* = 4.77, *p* = .001) across educational status. Overall, both national entitlement and SDO decreased as the educational level increased.

Additionally, a one-way ANOVA was performed to investigate whether the study variables differed across socioeconomic levels. The findings indicated that only SDO significantly varied across socioeconomic levels (*F* = 4.33, *p* = .005). Participants with higher socioeconomic status reported lower levels of SDO compared to those with lower socioeconomic status.

Following the group comparison analyses, the relationships among variables were examined. Pearson correlation analysis revealed that national entitlement was positively correlated with SDO (*r* = .24, *p* < .001) and negatively correlated with PSGC (*r* = −.22, *p* < .001). Similarly, SDO was negatively correlated with PSGC (*r* = −.44, *p* < .001) (see Table 3).

### Mediation analysis

**Fig. 1:**
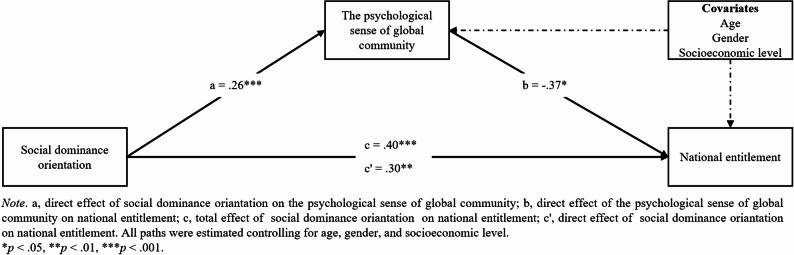
Mediation model with unstandardized coefficients controlling for age, gender, and socioeconomic level

Finally, a mediation analysis was run to reveal whether PSGC statistically mediates the relationship between SDO and national entitlement, while controlling for age, gender, and socioeconomic level. Table 4; Fig. 1 display the results of the tested mediation model. The findings indicated that SDO was significantly and negatively related to PSGC (*β* = -0.26, *t* = -8.34, *p* < .001), explaining 20% of the variance in PSGC. In addition, both SDO (*β* = 0.30, *t* = 2.89, *p* < .01) and PSGC (*β* = -0.37, *t* = -2.24, *p* < .05) were significantly correlated with national entitlement. Together, SDO and PSGC accounted for 11% of the total variance in national entitlement. Importantly, the indirect effect of SDO on national entitlement through PSGC was significant (*β* = 0.10, *SE* = 0.05, 95% CI [0.004, 0.210]), suggesting that PSGC may functions as a mediator in the relationship between SDO and national entitlement, alongside the direct relation of SDO (see Table 4).

**Table 4:** Unstandardized coefficients for the hypothesized mediation model controlling for age, gender, and socioeconomic level

Outcomes												
Antecedent	*M* (Psychological sense of global community)						*Y* (National entitlement)					
					95%						95%	
	*Coeff*	*SE*	*t*	*p*	LLCI	ULCI	*Coeff*	*SE*	*t*	*p*	LLCI	ULCI
*X* (Social dominance orientation)	-0.26	0.31	-8.34	0.000	-0.33	-0.20	0.30	0.11	2.89	0.004	0.10	0.51
*M* (Psychological sense of global community)	-	-	-	-	-	-	-0.37	0.17	-2.24	0.03	-0.70	-0.05
Constant	4.29	0.16	27.39	0.000	3.98	4.60	6.18	0.86	7.20	0.000	4.49	7.87
	*R²* = 0.20						*R²* = 0.11					
	*F* = 20.82 *p* < .001						*F* = 7.68 *p* < .001					

*SE* Standard error, *Coeff* unstandardized coefficient, *X* independent variable, *M* mediator variable, *Y* outcome variable

## Conclusions

The primary purpose of the current study was to adapt the Psychological Entitlement Scale to the national level and to examine the relationship of national entitlement with SDO and PSGC. Although the cross-sectional design and convenience sampling approach impose some limitations regarding causal interpretations and generalizability, this study contributes to the emerging literature on entitlement-based beliefs by providing initial psychometric evidence for the adaptation of psychological entitlement at the national level and by examining its relationship with intergroup ideological orientations. This approach is consistent with previous studies that have extended individual-level concepts to broader social contexts (e.g., de Zavala et al. [29]; Cai & Gries [20]), thereby supporting the conceptual and methodological validity of adapting psychological concepts to the national level.

Initially, the psychometric properties of the National Entitlement Scale, based on the Psychological Entitlement Scale [1], were examined, and findings from EFA and CFA indicated that the National Entitlement Scale was found to have a high internal consistency and a unidimensional, valid and reliable structure consisting of 8 items. Although Cai and Gries [20] considered national entitlement to be a dimension of national narcissism, they reported that their scale had an internal consistency ranging from 0.78 to 0.84 based on 6 items. Therefore, the findings of the present study are consistent with those of Cai and Gries [20].

In the present study, preliminary analyses indicated that SDO significantly differed by gender. Men were found to have higher SDO compared to women. This finding supports the theoretical and practical background [6, 59], indicating that SDO reflects tendencies to accept social inequality and hierarchy. Moreover, it is widely recognized that individuals high in SDO tend to endorse and maintain existing hierarchies [8]. Therefore, men’s higher SDO scores may imply that they tend to support traditionally more dominant, power-oriented, and authoritarian social structures. Accordingly, men may be more likely to take an active role in maintaining and sustaining social hierarchies.

Similarly, analyses indicated that PSGC significantly differed by gender. In the current study, women had higher PSGC scores. The difference in PSGC by gender suggests that individuals’ sense of commitment to the global community is influenced by broader sociocultural dynamics. In addition, women’s higher PSGC scores indicate that they tend to experience a stronger sense of global responsibility and moral concern. This tendency is in line with the findings of many previous studies [60, 61].

The findings also revealed that national entitlement and SDO decreased as the level of education increased. This may be because individuals with a higher education level are more likely to endorse less hierarchical social structures, perceive their nation as less superior, and show greater sensitivity to social equality and justice [6]. This finding suggests that highly educated individuals tend to have broader perspectives and a deeper understanding of global justice. In addition, this finding indicates that increasing the level of education may serve as a strategic means to reduce positive attitudes toward social inequality.

The Social Dominance Theory suggests that individuals with low socioeconomic status are more likely to exhibit hierarchical attitudes and to accept social inequality as a natural condition. On the other hand, it indicates that individuals with higher socioeconomic status are more likely to display egalitarian and less dominant attitudes because they have more resources and opportunities [8]. Consistent with this core idea of the theory, the findings of the present study demonstrated that the SDO scores of individuals with high socioeconomic status were lower. Individuals high in SDO perceive their group to be superior to others [12], and this orientation is associated with features that cause modern conflicts, including ethnocentrism [13] and nationalism [18]. In the present study, SDO was found to be positively correlated with national entitlement, which could be regarded as a construct associated with nationalism but conceptually distinct from. This suggests that individuals high in SDO are more likely to exhibit higher levels of national entitlement, and that when individuals embrace inequality and hierarchy between social groups, they also hold a belief that their nation should be entitled to various privileges. Thus, these findings are consistent with previous studies indicating that SDO is positively correlated with patriotism and nationalism [16] and national entitlement [20].

As mentioned earlier, SDO represents an attitude that supports the legitimization of inequalities between social groups [8]. Therefore, individuals with higher SDO tend to exhibit a stronger tendency to maintain social hierarchies and inequalities. On the other hand, PSGC describes perceiving oneself as equal to people all over the world [39, 40]. Thus, the finding of a negative relationship between SDO and PSGC in this study aligns with previous research.

Similarly, the present study demonstrated a negative correlation between PSGC and national entitlement. This finding implies that PSGC may attenuate individuals’ feelings of superiority and entitlement toward their nation. In other words, individuals who see themselves as part of a global community are likely to reduce perceptions that their nation should possess special entitlements. Although there are no studies directly investigating the relationship between these variables, this result is in the expected direction and is consistent with related literature. This is because PSGC refers to individuals’ perception of belonging not only to a national but also to a global community. On the other hand, it can be argued that individuals with higher national entitlement may weaken their ties with international communities, believing that their nation is entitled to greater privileges.

Another considerable result of the present study is that PSGC was significantly related to national entitlement and may function as a mediator in the relationship between SDO and national entitlement. This suggests that PSGC may be associated with the relationship between SDO and national entitlement. In other words, SDO was related to PSGC, which in turn was related to national entitlement, implying that PSGC may help explain the relationship between SDO and national entitlement. One possible way to understand this pattern is that PSGC reflects a form of human identity. According to Malsch [39], PSGC reflects identification with people from all over the world. Such a broad social identity may be related to greater acceptance of more egalitarian social structures rather than hierarchical ones and may also be associated with prioritizing the rights of all humanity over national interests, which may help explain its association with national entitlement in the context of SDO. In this respect, the findings of the current study are consistent with previous studies [62, 63], highlighting the positive intergroup effects of inclusive identities.

The finding regarding PSGC’s mediating role may also be related to Turkish cultural values. Türkiye is often characterized as relatively collectivist [64], with cultural orientations that emphasise relational values and interdependence [65]. Such cultural dynamics may, on the one hand, be associated with stronger national identity and potentially higher levels of national entitlement, and on the other hand, be associated with greater sensitivity to social cohesion and concern for the broader human community. These relational values may be related to the perceived importance of PSGC as a culturally meaningful mechanism associated with lower acceptance of hierarchy-supporting attitudes. Therefore, although national entitlement and PSGC may have a negative relationship at the individual level, the broader cultural context can support both orientations simultaneously, potentially creating a social environment in which PSGC is linked with lower levels of hierarchy-supporting tendencies associated with SDO.

### Limitations and future directions

The present study has several fundamental limitations. First, the study relied on self-report data. As is well known, in self-reported measures, participants are more likely to respond in a socially desirable manner. To overcome this limitation, future studies should employ behavioral and implicit measures. The analyses were based on cross-sectional data collected from volunteers. To strengthen the generalizability of the results and gain a deeper understanding of the relationships between the variables, it is recommended that future studies employ experimental or longitudinal designs that include individuals without internet access. Another limitation stems from the method used to recruit participants for the study. Using convenience sampling can lead to potential biases, as the sample may not be representative of the broader population, limiting generalizability. Moreover, self-selection bias may occur because individuals who are more active on social media or more motivated to participate may differ systematically from non-participants, potentially impacting the study’s internal validity. These limitations should be considered when interpreting the findings.

Another notable limitation is related to the scope of the concepts included in this study. Although the main objective of the study is to adapt the Psychological Entitlement Scale at the national level and to validate its validity, related concepts such as national grandiosity, patriotism, nationalism, and chauvinism have not been included in the study. Cai and Gries [20] conceptualised national entitlement as a dimension of national narcissism, in which national grandiosity is an essential component. National entitlement was also found to be positively correlated with patriotism, nationalism, and chauvinism. The absence of these measures in the current study limits the ability to empirically assess the distinguishability of national entitlement from these related concepts. Future research could build on the current findings by including national grandiosity, patriotism, nationalism, and chauvinism. This would allow for a more comprehensive assessment of the National Entitlement Scale’s discriminant validity and further clarify the unique contribution and added value of the National Entitlement Scale.

Another methodological limitation concerns the use of a single dataset that was randomly divided into two subgroups for the EFA and the CFA. While this approach is commonly employed in psychometric validity studies in the absence of separate samples (e.g [48]), , using entirely separate data collections to conduct EFA and CFA would provide stronger evidence for the stability and generalisability of the factor structure. Therefore, future research could aim to replicate the current findings with independent participant groups to further confirm the robustness of the psychometric properties of the National Entitlement Scale.

In this study, the Psychological Entitlement Scale, developed by Campbell et al. [1], was adapted to the national context of Türkiye, as shown in Appendix 1. According to previous research, relational values tend to be more dominant than individual values in Turkish culture [64, 65]. Therefore, it is reasonable to assume that national entitlement, closely associated with national identity, is particularly salient in Türkiye and other cultures where relational values prevail. However, examining whether the National Entitlement Scale yields similar psychometric properties in more individualistic cultures could provide valuable insights into the relationship between national entitlement and cultural context, thereby contributing significantly to the relevant field. Thus, future research should aim to establish the cross-cultural reliability of the National Entitlement Scale by confirming its validity in different cultural contexts. To facilitate such cross-cultural research, the American version of the National Entitlement Scale is presented in Appendix 2 for studies aiming to examine whether the scale exhibits comparable psychometric properties in American culture, where individual values are more prominent than in Türkiye and where English is the formal/primary language.

The National Entitlement Scale provides valuable opportunities to examine how individual-level psychological entitlement tendencies interact with national identity, cultural values, and nationalism. This study has revealed that the National Entitlement Scale can serve as a useful measurement tool, particularly for researchers studying intergroup relations. Using the National Entitlement Scale, researchers can examine the impact of individuals’ perceptions of their national identity on contemporary global issues, such as social belonging, nationalism, ethnocentrism, and political attitudes. Moreover, the scale is especially relevant for researchers exploring power dynamics between social groups, as it enables a deeper understanding of the social-psychological consequences of perceptions of social justice and equality.

The current research also demonstrated that SDO was correlated with national entitlement both directly and indirectly through PSGC. Nevertheless, investigating how broader psychological and societal factors influence this relationship represents an important direction for future research. In particular, examining how changing social conditions over time impact this relationship could provide valuable insights. Furthermore, future studies examining how the relationships among the variables used in this study differ across sociodemographic and cultural contexts would facilitate a deeper understanding of these psychological factors and may inform the development of more targeted, culturally sensitive intervention strategies based on PSGC. Future cross-cultural studies would be particularly insightful in elucidating how cultural values shape the relationships between SDO, PSGC, and national entitlement. For instance, comparing individualistic and collectivist cultures could reveal whether PSGC’s mediating role functions similarly across different cultural contexts.

It was observed that only PSGC mediated the relationship between SDO and national entitlement after controlling for age, gender, and socioeconomic level. However, multiple psychological constructs may potentially have a mediating role in this relationship. Therefore, future studies could explore additional psychological mechanisms, such as identification with all humanity, empathy, or compassion, to better understand the underlying processes of hierarchy-supporting attitudes and national entitlement. Furthermore, given that the PSGC can attenuate the impact of the SDO on national entitlement, educational policies and social programs could be designed to enhance young people’s understanding of global responsibilities, human rights, social equality, and justice. Thus, a global social identity might contribute to a more inclusive and equitable understanding of national entitlement.

In conclusion, the results revealed that the short and unidimensional version of the National Entitlement Scale offers a reliable and valid tool for assessing entitlement at the national level. Furthermore, the results suggested that PSGC may be negatively correlated with national entitlement and may be associated with a weaker relationship between SDO and national entitlement, highlighting its importance for understanding intergroup hostility.

## Data Availability

The dataset generated during and/or analyzed during the present study is available from the corresponding author upon reasonable request.

## Appendix 1

### Turkish version of the National Entitlement Scale

Lütfen aşağıdaki ifadelere kendi görüşlerinizi en iyi yansıtan sayıyı kullanarak yanıt verin. Lütfen aşağıdaki 7’li ölçeği kullanın:

1 = kesinlikle katılmıyorum.

2 = katılmıyorum.

3 = kısmen katılmıyorum.

4 = kararsızım.

5 = kısmen katılıyorum.

6 = katılıyorum.

7 = kesinlikle katılıyorum.

1. Dürüst olmak gerekirse, Türkiye’nin diğer ülkelerin hakkettiklerinden daha fazlasını hak ettiğini düşünüyorum.
2. Önemli şeyler Türkiye’nin olmalı.
3. Türkiye için en iyiyi isterim çünkü buna layık.
4. Uluslararası camiada Türkiye’nin özel bir muamele görmesi gerekmez. (ters kodlanmış ifade)
5. Türkiye sahip olduklarından daha fazlasını hak ediyor.
6. Türkiye gibi ülkeler ara sıra fazladan bir şansı hak eder.
7. İşler Türkiye’nin istediği gibi gitmeli.
8. Türkiye’nin her şeyin daha fazlasını hak ettiğini düşünüyorum.

## Appendix 2

### English version of the National Entitlement Scale

Please respond to the following items using the number that best reflects your own beliefs. Please use the following 7-point scale:

1 = strong disagreement.

2 = moderate disagreement.

3 = slight disagreement.

4 = neither agreement nor disagreement.

5 = slight agreement.

6 = moderate agreement.

7 = strong agreement.

1. I honestly feel that America is just more deserving more than other countries.
2. Great things should come to America.
3. I demand the best for America because America’s worth it.
4. America does not necessarily deserve special treatment in the international community. (reverse-coded item)
5. America deserves more than it has.
6. Countries like America deserve an extra break now and then.
7. Things should go America’s way.
8. America is entitled to more of everything.

## Abbreviations

AGFI: Adjusted goodness-of-fit index
CFA: Confirmatory factor analysis
CFI: Comparative fit index
EFA: Exploratory factor analysis
GFI: Goodness-of-fit index
NFI: Normed fit index
PSGC: Psychological sense of global community
RMSEA: Root mean square error of approximation
SDO: Social dominance orientation
SRMR: Standardized root mean squared residual
χ2/df: Chi-square/degrees of freedom
